# Genomic detection of a virus lineage replacement event of dengue virus serotype 2 in Brazil, 2019

**DOI:** 10.1590/0074-02760190423

**Published:** 2020-05-15

**Authors:** Jaqueline Goes de Jesus, Karina Rocha Dutra, Flavia Cristina da Silva Sales, Ingra Morales Claro, Ana Carolina Terzian, Darlan da Silva Candido, Sarah C Hill, Julien Thézé, Celeste Torres, Tatiana Lang D’Agostini, Alvina Clara Felix, Andreia F Negri Reis, Luiz Carlos Junior Alcantara, André L de Abreu, Júlio HR Croda, Wanderson K de Oliveira, Ana Maria Bispo de Filipis, Maria do Carmo Rodrigues dos Santos Camis, Camila Malta Romano, Nick J Loman, Oliver G Pybus, Ester Cerdeira Sabino, Mauricio L Nogueira, Nuno Rodrigues Faria

**Affiliations:** 1 Universidade de São Paulo, Instituto de Medicina Tropical, São Paulo, SP, Brasil; 2 Faculdade de Medicina de São José do Rio Preto, Laboratório de Pesquisa em Virologia, São José do Rio Preto, SP, Brasil; 3 University of Oxford, Department of Zoology, Oxford, United Kingdom; 4 Fundação Oswaldo Cruz-Fiocruz, Instituto Oswaldo Cruz, Laboratório de Flavivírus, Rio de Janeiro, RJ, Brasil; 5 Secretaria de Estado da Saúde, Centro de Vigilância Epidemiológica Professor Alexandre Vranjac, Coordenadoria de Controle de Doenças/São Paulo, SP, Brasil; 6 Secretaria Municipal de Saúde, São José do Rio Preto, SP, Brasil; 7 Universidade Federal de Minas Gerais, Belo Horizonte, MG, Brasil; 8 Ministério da Saúde, Secretaria de Vigilância em Saúde, Coordenação Geral de Laboratórios de Saúde Pública, Brasília, DF, Brasil; 9 Universidade Federal da Grande Dourados, Laboratório de Pesquisa em Ciências da Saúde, Dourados, MS, Brasil; 10 Fundação Oswaldo Cruz-Fiocruz, Campo Grande, MS, Brasil; 11 Universidade de São Paulo, Faculdade de Medicina, Hospital das Clínicas, São Paulo, SP, Brasil; 12 University of Birmingham, School of Biosciences, Institute of Microbiology and Infection, United Kingdom

**Keywords:** dengue 2, outbreak, genomic surveillance, lineage replacement, Brazil

## Abstract

**BACKGROUND:**

Despite efforts to mitigate the impact of dengue virus (DENV) epidemics, the virus remains a public health problem in tropical and subtropical regions around the world. Most DENV cases in the Americas between January and July 2019 were reported in Brazil. São Paulo State in the southeast of Brazil has reported nearly half of all DENV infections in the country.

**OBJECTIVES:**

To understand the origin and dynamics of the 2019 DENV outbreak.

**METHODS:**

Here using portable nanopore sequencing we generated20 new DENV genome sequences from viremic patients with suspected dengue infection residing in two of the most-affected municipalities of São Paulo State, Araraquara and São José do Rio Preto. We conducted a comprehensive phylogenetic analysis with 1,630 global DENV strains to better understand the evolutionary history of the DENV lineages that currently circulate in the region.

**FINDINGS:**

The new outbreak strains were classified as DENV2 genotype III (American/Asian genotype). Our analysis shows that the 2019 outbreak is the result of a novel DENV lineage that was recently introduced to Brazil from the Caribbean region. Dating phylogeographic analysis suggests that DENV2-III BR-4 was introduced to Brazil in or around early 2014, possibly from the Caribbean region.

**MAIN CONCLUSIONS:**

Our study describes the early detection of a newly introduced and rapidly-expanding DENV2 virus lineage in Brazil.

Over 400 million people are estimated to be at risk of acquiring dengue virus (DENV - genus *Flavivirus*, family *Flaviviridae*),^1^ a mosquito-borne virus transmitted in tropical and subtropical areas by competent urban vectors such as the mosquitoes *Aedes aegypti* and *Aedes albopictus*.^2^ DENV is classified into four distinct virus lineages named serotypes 1 to 4 (DENV1-4). Within each DENV serotype there is some degree of genetic variation, and at least 19 DENV genotypes have now been described.^3^ Increasing human mobility has facilitated the co-circulation of multiple dengue serotypes in the same region,^4^ a pattern known as hyperendemicity. In such regions, DENV epidemiological dynamics are complex and typically characterised by virus genotype replacement every 7-10 years.^5^ ^,^ ^6^ ^,^ ^7^ ^,^ ^8^ ^,^ ^9^ Clade replacement is typically associated with an increased number of cases and cases with severe disease. Certain genotypes and lineages seem to be more frequently associated with severe disease outcomes.^8^ ^,^ ^10^ ^,^ ^11^

DENV was first detected in Brazil in 1982.^12^ Since then, it has become a serious public health concern due to its high incidence in the country and association with severe dengue illnesses.^13^ Co-circulation of dengue serotypes has been observed throughout Brazil,^14^ ^,^ ^15^ particularly in highly populated areas of the Southeastern region that includes the federal states of São Paulo, Rio de Janeiro, Minas Gerais and Espírito Santo. Between 1995 and 2015, Brazil reported nearly 8 million DENV cases, which comprises 55% of all cases reported in the Americas during this period.^12^ Over the last thirty years, the Southeast region of Brazil has reported 2225 dengue-related fatal cases, representing 43% of all dengue-related deaths in the country.^13^

In the first half of 2019 (1st Jan to 30th Jun) Brazil has already reported 1,127,244 dengue cases.^12^ Importantly, this number is nearly 8-fold higher than in the previous year and corresponds to 89% of all the dengue cases reported in the Americas over the same period. The number of severe cases (n = 710) and dengue-related deaths (n = 366) also increased by at least 2.3-fold in comparison with 2018.

The Southeast region of Brazil has reported 65.7% of all dengue cases identified in the country.^16^ São Paulo state is the most highly densely populated state and the main socio-economic hub in Brazil; previous studies suggest the state was an important source location for the spread of DENV4 in the country.^17^ Here, we characterise the genetic diversity of circulating DENV in two municipalities of São Paulo State. We generated virus genome sequences from the ongoing outbreak using a well-established portable genomic approach.^18^ ^,^ ^19^ In an attempt to better understand the origin and dynamics of the 2019 outbreak, we conducted comprehensive genetic analyses to understand the relationship between the current epidemic strains and those that circulated in previous outbreaks in the Americas.

## MATERIALS AND METHODS

Brazil is organised into 26 federal states and one Federal District. Sao Paulo State is the most populous Brazilian state and comprises 615 municipalities. São José do Rio Preto is the 11th most populated municipality (450,657 inhabitants), and Araraquara the 32nd most populated municipality (230,770 inhabitants) in the state (www.ibge.gov.br). In each municipality, the number of dengue suspected cases is notified by local public health secretaries to the Centro de Vigilância Epidemiológica Prof Alexandre Vranjac (CVE), part of São Paulo’s State Health Secretary [Supplementary data **(Table I)**]. As part of dengue surveillance efforts in São Paulo State, samples are collected from patients suspected of acute DENV infection and tested for DENV by real-time quantitative reverse transcription polymerase chain reaction (qRT-PCR) by several research centres and public health institutions, including Adolfo Lutz Institute. Monthly numbers of dengue cases per serotype are then aggregated by CVE.

To assess the genetic diversity of dengue cases circulating in São Paulo State, we selected 20 qRT-PCR positive samples DENV serotype 2 from patients in two municipalities, Araraquara and São José do Rio Preto. The majority of the samples (19 out of 20) was collected between January and the end of April 2019; however, to investigate whether the same lineage was circulating before 2019, we also included one sample from São José do Rio Preto collected in early June 2017. Samples had mean RT-qPCR cycle-threshold values of 19.8 (range: 16.4 - 25). Diagnostic details and symptoms are shown in Supplementary data **(Table II)**].

Residual anonymised clinical diagnostic samples from Araraquara were obtained following ethical approval by Hospital das Clínicas - University of São Paulo’s Institutional Review Board (CAPPesq) (number 3.156.894). São José do Rio Preto samples were obtained from virological surveillance routine, within the study approved by University of São José do Rio Preto Institutional Review Board approval #48982/2012. We used residual anonymised clinical diagnostic samples, with no risk to patients, which were provided for research and surveillance purposes within the terms of Resolution 510/2016 of CONEP (Comissão Nacional de Ética em Pesquisa, Ministério da Saúde; National Ethical Committee for Research, Ministry of Health).

The 20 qRT-PCR-positive DENV2 samples were subjected to viral genomic amplification at the Institute of Tropical Medicine, University of São Paulo, Brazil. Genome sequencing was conducted using the portable nanopore MinION sequencing platform, which has been used previously in Brazil during outbreaks of Zika virus and yellow fever virus.^9^ ^,^ ^10^ ^,^ ^11^ Sequencing was performed using a multiplex PCR primer scheme designed to amplify the entire coding region of DENV2 as previously described.^12^

RNA was extracted and reverse-transcribed to cDNA using Superscript IV First-Strand Synthesis System (Thermo Fisher Scientific, MA, USA) and random hexamer priming. Then, multiplex PCR was performed to generate overlapping amplicons of the whole genome of the targeted DENV2 strain. DENV2 genome amplification consisted of 35 cycles of PCR according to the reaction mix and thermocycling described by Quick et al.^19^ AmpureXP purification beads (Beckman Coulter, High Wycombe, UK) were used to clean up PCR products, which were then quantified by Qubit dsDNA High Sensitivity assay on a Qubit 3.0 instrument (Life Technologies). Sequencing libraries were generated using the Genomic DNA Sequencing Kit SQK-LSK108 (Oxford Nanopore Technologies), by pooling, in equimolar proportions, a total of 250 ng of PCR products previously barcoded using the Native Barcoding Kit (NBD103, Oxford Nanopore Technologies, Oxford, UK). The libraries were loaded onto an Oxford Nanopore flow cell R9.4 (FLO-MIN106) and sequencing data were collected for 30 h. The median number of mapped reads was 45,013 reads per sample, and the generated consensus genomes had a mean coverage of 81% of the genome at 20x minimum sequencing depth. Sequencing statistics for each sample are shown in Supplementary data **(Table II)**.

To investigate the origins of the newly generated genomes we downloaded all DENV2 nucleotide sequences longer than 1400 nucelotides (nt) from GenBank^16^ that had a known date (year, and month and day when available) and location (country, and city when available; n = 1630 as of 20 June 2019). We aligned these sequences using MAFFT automatic settings^18^ and manually edited them with AliView v1.19.^19^ We subsequently constructed an initial maximum likelihood phylogeny to help identify the genotypes of strains that have historically circulated (collected before 2019) and are currently circulating (collected in 2019) in Brazil. For this genotype assessment, we constructed phylogenies using FastTree v.2 with gamma-distributed among site rate heterogeneity and a general time reversible nucleotide substitution model.^20^ We observed that all sequences from the Americas (including the newly generated sequences) grouped together in a well-supported monophyletic clade. Therefore, we next constructed a dataset comprising only sequences collected in the Americas (n = 670).

**Fig. 1:**
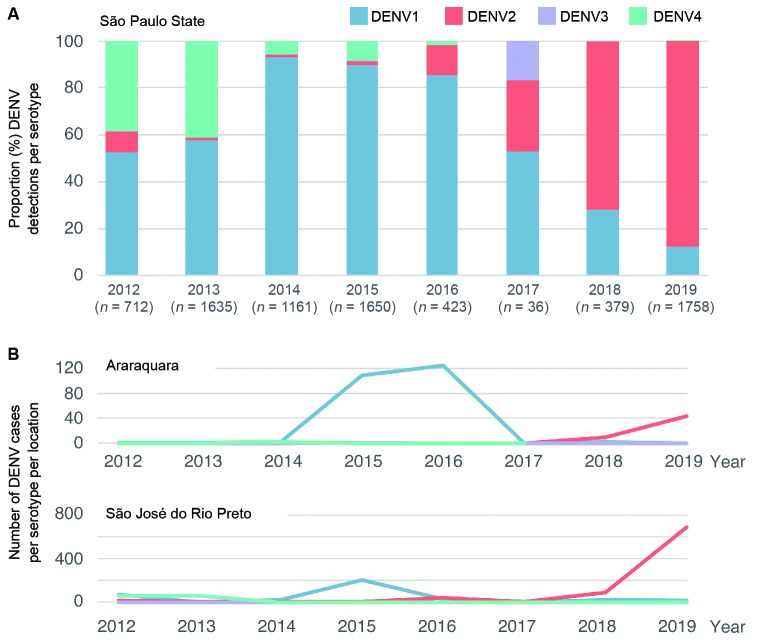
annual number of dengue virus (DENV) cases by serotype reported to the Centro de Vigilância Epidemiológica (CVE), São Paulo State, Brazil, between January 2012 and June 2019. (A) Proportion (percentage) of dengue cases by serotype reported in São Paulo State (total n = 7754). (B) Number of dengue cases by serotype in Araraquara (total n = 294) and in São José do Rio Preto (total n = 1347).

To reduce sampling bias towards a high number of samples from well-sampled countries, we removed duplicate sequences (same day and location) from Nicaragua and Peru, yielding a final dataset of 436 genomes (including 66 genomes collected in Brazil between 1990 to 2013). Maximum likelihood phylogenies of the American DENV2 genomes (n = 670 and n = 436) were generated using PhyML^20^ available through Seaview v.4.6.1,using gamma-distributed among site rate heterogeneity and a general time reversible nucleotide substitution model.^21^ Root-to-tip divergence and temporal signal was evaluated using TempEst^21^ [Supplementary data **(Fig. 1)**].

Georefenced and time-stamped phylogenies were constructed using a discrete phylogeographic approach as previously described.^19^ ^,^ ^22^ In brief, countries were grouped into four geographic regions consisting of Brazil (n = 86), Central America and Mexico (n = 45), South America (n = 132) and Caribbean (n = 173). Inferred locations at each internal node and corresponding dated phylogenies trees were estimated using BEAST1.10.^23^ MCMC convergence was inspected using Tracer.v1.7 and summary trees were generated using TreeAnnotator.^23^

*Ethics* - Samples were provided for research and surveillance purposes within the terms of Resolution 510/2016 of CONEP (National Ethical Committee for Research, Ministry of Health).

## RESULTS AND DISCUSSION

Epidemiological information on the number of confirmed dengue cases with associated serotype information shows that all four dengue serotypes co-circulate in Sao Paulo State (Fig. 1A). While in 2012 DENV1 (52%, n = 373/712) and DENV4 (39%, n = 274/712) predominated, DENV 2 (9%, n = 64/712) and DENV3 (0.1%, n = 1/712) were also detected. Since 2014 a notable increase in DENV2 cases is observed, with frequencies increasing rapidly from 1.03% (n = 12/1161) in 2014, to 1.39% (n = 23/1650) in 2015, 12.53% (n = 53/423) in 2016, 30.56% (n = 52/423) in 2017, 71.5 (n = 271/379) in 2018 to 87.5% (n = 1539/1758) in the first semester of 2019 (Fig. 1A).

We next report genomic epidemiological findings from our surveillance of two municipalities in the São Paulo State, Araraquara (ARA) and São José do Rio Preto (SJRP), between early June 2017 and the end of April 2019. In Araraquara, a total of 96% (n = 52/54) dengue cases notified to CVE were caused by DENV2 between 2017 and June 2019; in São José do Rio Preto, during the same period, 95% (n = 779/821) were caused by this serotype (Fig. 1B). From each location, 10 RT-qPCR positive samples (mean cycle threshold: 19.8, range: 16.4 to 25) were randomly selected for complete genome sequencing using a previously developed amplicon-based approach for on-site sequencing using the minION platform.^24^ ^,^ ^25^

All sequenced samples were classified as DENV2 using an automated phylogenetic-based serotyping tool.^3^ To investigate the genotypic diversity and the origins of the ongoing DENV2 outbreak in Brazil, we performed phylogenetic analysis of DENV2 using all publicly available complete or partial DENV2 genome sequences (n = 1,630 as of 21 June 2019).

**Fig. 2:**
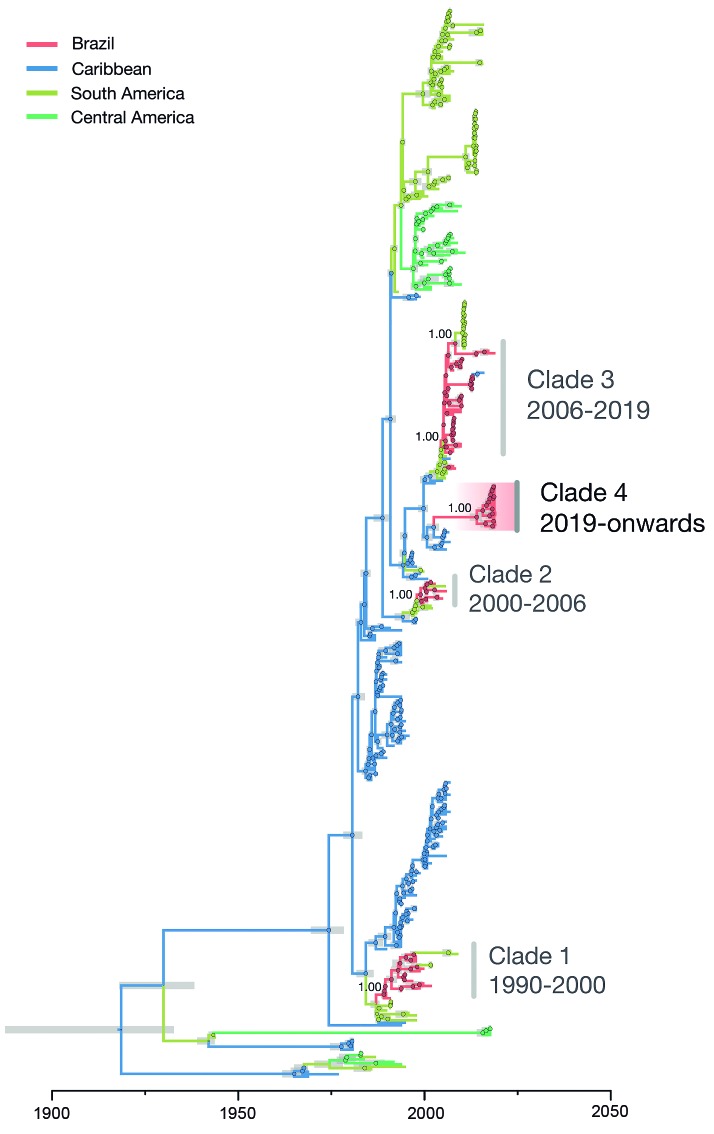
evolutionary history of dengue virus serotype 2 (DENV2) in Brazil. Bayesian dated phylogeographic tree of DENV serotype 2 (n = 426) in the Americas. New clade comprising isolates from 2019 collected in Araraquara and São José do Rio Preto (São Paulo State) are highlighted with a red gradient. 95% Bayesian credible intervals for node ages are shown for nodes with posterior support above 0.95.

DENV2 is classified into six genotypes, named I-VI.^3^ To date, three genetic lineages of DENV2 genotype III (DENV2-III) have been reported in Brazil on the basis of phylogenetic analysis of the relationships of partial and complete genomes of circulating strains. These three circulating lineages have been named as lineages 1-3^5^ or BR1-BR3.^26^ Our maximum likelihood (ML) phylogenetic analysis shows a separate introduction of DENV2, hereafter named DENV2-III BR-4 [Supplementary data **(Fig. 2)**].

Our phylogenetic analysis further shows that virus genomes recovered from the 2019 cases all belong to genotype III (also known as American/Asian genotype). Notably, our analysis strongly supports (approximate likelihood ratio test = 1.00) the clustering of the2019 DENV2 cases from Brazil (18 of the 19 sequences collected in 2019) into a single monophyletic group (named here as DENV2-III BR-4), which is the result of a new and recent introduction of DENV2-III from outside of Brazil [Supplementary data **(Fig. 2)]**. In addition, two sequences from São José do Rio Preto, one sampled in June 2017 (ID: 126) and another in January 2019 (ID: 140) grouped with the isolates from a previous clade circulating since 2006 in the Southeast region (BR-3).

To investigate in more detail the origins of the new DENV2-III BR-4 circulating in Brazil in 2019, we conducted a statistical phylogeographic analysis of 436DENV2 genome sequences representing DENV2 genotypes circulating in the Americas. Our analysis reveals that the DENV2-III BR-4lineage was introduced in or around 2014 (95% Bayesian credible interval: 2012 to 2015) [Supplementary data **(Fig. 2)**]. From then onwards, the proportion of DENV2 cases in São Paulo State increased (Fig. 1A). Estimation of the ancestral location of this lineage reveals that it likely originated in the Caribbean region (posterior support = 1.00). However, we note that the new lineage could have also been introduced from countries with tropical climates in South America (e.g. French Guiana or Suriname) from where no recent virus genomic data has been made available. Molecular clock analyses have shown that novel DENV lineages have been introduced in Brazil every 7 to 10 years, after which they are replaced by a novel lineage introduced from other locations. For example, DENV2-IIIBR-1 was introduced in 1990, DENV2-III BR-2 in 1998, DENV2-III BR-3 in 2005.^5^ Given that the former lineage replacement in Brazil resulted from a strain introduced around 2005, our dating estimate for the introduction of DENV2-III BR-4 and the noticeable increase in number of DENV2 cases in Sao Paulo State are indicative of a new DENV2 lineage replacement in Brazil.

Our data reveals that two distinct virus lineages (DENV2-III BR-3 and DENV2-III BR-4) are co-circulating in a single location (São José do Rio Preto) (Fig. 2). Although we are limited by the small sample size of the data analysed here, it is remarkable that the frequency of DENV2-III 2019 strains belonging to BR-3 is 5.3% (n = 1/19) and for BR-4 is 94.7% (n = 18/19). The upsurge in the number of dengue and dengue severe cases observed in São Paulo State, combined with the simultaneous detection of these two lineages and the increased frequency of BR-4 suggests that we are retrospectively reporting a lineage replacement event in São Paulo State. Additional data from other Brazil locations and from the Caribbean will allow us to reduce uncertainty in the dating estimates and to quantify the extent of the geographic spread of the DENV2-III BR-4 lineage reported here.

Although our genetic analysis estimates date of the introduction of the DENV2-III BR-4 lineage at least four years before its detection in 2019 (Fig. 2), and it is in agreement with the low prevalence of DENV2 cases in São Paulo State in 2014 (Fig. 1A), more genetic end epidemiological data from 2014 to 2018 are necessary to precise the date of introduction and study how this lineage is stablishing in the State. Low viral lineage prevalence could explain the undetected circulation of DENV2-III BR-4 in São Paulo during a period when Brazil was hit by explosive epidemics of Zika, chikungunya and yellow fever viruses.^27^ ^,^ ^28^ This highlights the need of improved longitudinal genomic surveillance using, for instance, sequence-independent approaches for arbovirus detection. Such approaches will be particularly critical in geographic regions with year-round DENV transmission in *Aedes* spp. mosquitoes, such as locations with tropical and subtropical climates.^2^ In conclusion, this study highlights the potential of integration of routine real-time genomic surveillance to better understand the arrival and the establishment of new virus lineages and other pathogens in the Americas.

*Data availability* - XML files and datasets analysed in this study are available in the GitHub repository (https://github.com/CADDECentre/DENV2-PILOT). New sequences have been deposited in GenBank under accession numbers 959467 - 959485 [Supplementary data (Table III)]. Raw sequence data have been deposited in SRA database from NCBI under numberPRJNA606238.
