# Supplementary material for: Genomic detection of a virus lineage replacement event of dengue virus serotype 2 in Brazil, 2019
Source: Mem Inst Oswaldo Cruz. 2020 May 15;115:e190423. doi: 10.1590/0074-02760190423 (PMC7227788; doi:10.1590/0074-02760190423)
Supplement: Supplementary file 1 [file 1678-8060-mioc-115-e190423-s.pdf]

Mem Inst Oswaldo Cruz, Rio de Janeiro, Vol. *114*: e190423, 2019

Brief description of dengue surveillance efforts in São Paulo State, Brazil

Brazil is organised into 26 federal states and one Federal District. São Paulo State is the most populous Brazilian State and comprises 615 municipalities. São José do Rio Preto is the 11th most populated municipality (450,657 inhabitants), and Araraquara the 32nd most populated municipality (230,770 inhabitants) in the State (www.ibge.gov.br). In each municipality, the number of dengue suspected cases is notified by local public health secretaries to the Centro de Vigilância Epidemiológica Prof Alexandre Vranjac (CVE), part of São Paulo's State Health Secretary. As part of dengue surveillance efforts in São Paulo State, samples are collected from patients suspected of acute dengue virus (DENV) infection and tested for DENV by real-time quantitative reverse transcription polymerase chain reaction (qRT-PCR) by several research centres and public health institutions, including Adolfo Lutz Institute. Monthly numbers of dengue cases per serotype are then aggregated by CVE are shown in Table I.

TABLE I

Number of dengue virus (DENV) serotyped cases available from the Vigilância Epidemiológica Prof Alexandre Vranjac (CVE) centre of the São Paulo's State Health Secretary.

		Year of symptoms							
Municipality/state	Serotype	2012	2013	2014	2015	2016	2017	2018	2019
ARA	DENV 1	373	941	1082	1482	362	19	107	216
	DENV 2	64	22	12	23	53	11	271	1539
	DENV 3	1	0	0	4	0	6	1	1
	DENV 4	274	672	67	141	8	0	0	1
SJRP	DENV 1	373	941	1082	1482	362	19	107	216
	DENV 2	64	22	12	23	53	11	271	1539
	DENV 3	1	0	0	4	0	6	1	1
	DENV 4	274	672	67	141	8	0	0	1
São Paulo State	DENV 1	373	941	1082	1482	362	19	107	216
	DENV 2	64	22	12	23	53	11	271	1539
	DENV 3	1	0	0	4	0	6	1	1
	DENV 4	274	672	67	141	8	0	0	1

ARA: Araraquara; SJRP: São José do Rio Preto; SP: São Paulo State.

cc BY

Residual anonymised clinical diagnostic samples

To assess the genetic diversity of dengue cases circulating in São Paulo State, we selected 20 qRT-PCR positive samples DENV serotype 2 from patients in two municipalities, Araraquara and São José do Rio Preto. The majority of the samples (19 out of 20) was collected between January and the end of April 2019; however, to investigate whether the same lineage was circulating before 2019, we also included one sample from São José do Rio Preto collected in early June 2017. Samples had mean RT-qPCR cycle-threshold values of 19.8 (range: 16.4 - 25). Diagnostic details and sequences stats are shown in Table II.

TABLE II

Available epidemiological data and sequencing statistics for samples analysed in this study

ID	municipality	Gender	Age	Sampling date	CT-value	No. reads mapped	Average depth coverage	Coverage (20x, %)
4000	ARA	F	17	05/02/2019	19.16	72982	3697.06	92.54
4011	ARA	M	16	06/02/2019	23.3	76613	3481.58	92.51
4013	ARA	F	17	06/02/2019	22.3	59563	3130.47	91.98
4019	ARA	M	15	06/02/2019	20.08	81386	3880.94	92.53
4020	ARA	F	19	07/02/2019	16.5	69446	3011.08	91.93
4112	ARA	M	15	08/02/2019	18.3	78543	3833.26	92.54
4210	ARA	F	14	15/02/2019	16.4	58396	3463.58	92.54
4213	ARA	F	19	16/02/2019	16.8	43137	2889.44	92.48
4216	ARA	M	17	16/02/2019	18.5	46889	1613.3	33.91
4105	ARA	F	20	08/02/2019	18.19	52037	3294.55	92.53
4218	ARA	M	19	21/02/2019	21.7	79264	3700.89	92.54
126	SJRP	-	-	07/06/2017	25	2494	276.67	60.26
137	SJRP	-	-	10/01/2019	21.4	2211	210.462	40.34
138	SJRP	-	-	13/01/2019	17.3	2644	199.651	79.46
140	SJRP	-	-	17/01/2019	21	4905	420.513	87.44
142	SJRP	-	-	18/01/2019	18.4	5526	428.731	82.29
143	SJRP	-	-	08/04/2019	18.9	5177	413.238	81.29
144	SJRP	-	-	29/04/2019	20.7	3266	262.355	74.23
145	SJRP	-	-	28/04/2019	17.5	2663	222.407	81.25
146	SJRP	-	-	15/04/2019	21.7	3659	304.34	74.22

ID: identification number; CT: RT-qPCR cycle threshold; ARA: Araraquara; SJRP: São José do Rio Preto.

Ethical approvals

Residual anonymised clinical diagnostic samples from Araraquara were obtained following ethical approval by Hospital das Clínicas - University of São Paulo's Institutional Review Board (CAPPesq) (number 3.156.894). São José do Rio Preto samples were obtained from virological surveillance routine, within the study approved by University of São José do Rio Preto Institutional Review Board approval #48982/2012. We used residual anonymised clinical diagnostic samples, with no risk to patients, which were provided for research and surveillance purposes within the terms of Resolution 510/2016 of CONEP (Comissão Nacional de Ética em Pesquisa, Ministério da Saúde; National Ethical Committee for Research, Ministry of Health).

Genome sequencing using Oxford Nanopore Technology

The 20 qRT-PCR–positive DENV2 samples were subjected to viral genomic amplification at the Institute of Tropical Medicine, University of São Paulo, Brazil. Genome sequencing was conducted using the portable nanopore MinION sequencing platform, which has been used previously in Brazil during outbreaks of Zika virus and yellow fever virus.[9,10,11] Sequencing was performed using a multiplex PCR primer scheme designed to amplify the entire coding region of DENV2 as previously described.[12]

Extraction of viral RNA

RNA was extracted and reverse-transcribed to cDNA using Superscript IV First-Strand Synthesis System (Thermo Fisher Scientific, MA, USA) and random hexamer priming. Then, multiplex PCR was performed to generate overlapping amplicons of the whole genome of the targeted DENV2 strain. DENV2 genome amplification consisted of 35 cycles of PCR according to the reaction mix and thermocycling described by Quick et al.(1) AmpureXP purification beads (Beckman Coulter, High Wycombe, UK) were used to clean up PCR products, which were then quantified by Qubit dsDNA High Sensitivity assay on a Qubit 3.0 instrument (Life Technologies). Sequencing libraries were generated using the Genomic DNA Sequencing Kit SQK-LSK108 (Oxford Nanopore Technologies), by pooling, in equimolar proportions, a total of 250 ng of PCR products previously barcoded using the Native Barcoding Kit (NBD103, Oxford Nanopore Technologies, Oxford, UK). The libraries were loaded onto an Oxford Nanopore flow cell R9.4 (FLO-MIN106) and sequencing data were collected for 30 h. The median number of mapped reads was 45,013 reads per sample, and the generated consensus genomes had a mean coverage of 81% of the genome at 20x minimum sequencing depth. Sequencing statistics for each sample are shown in Table II. Raw and processed data are available on GitHub (https://github.com/CADDECentre/DENV2-PILOT).

Genetic analysis

To investigate the origins of the newly generated genomes we downloaded all DENV2 complete or near-complete genomes longer than 8500 nucleotides (nt) from GenBank(2) that had a known date (year, and month and day when available) and location (country, and city when available; n = 1630 as of 20 June 2019). We aligned these sequences using MAFFT automatic settings(3) and manually edited them with AliView v1.19.(4) We subsequently constructed an initial maximum likelihood phylogeny to help identify the genotypes of strains that have historically circulated (collected before 2019) and are currently circulating (collected in 2019) in Brazil. For this genotype assessment, we constructed phylogenies using FastTree v.2 with gamma-distributed among site rate heterogeneity and a general time reversible nucleotide substitution model.(5) We observed that all sequences from the Americas (including the newly generated sequences, Table III) grouped together in a well-supported monophyletic clade. Therefore, we next constructed a dataset comprising only sequences collected in the Americas (n = 670). To reduce sampling bias towards a high number of samples from well-sampled countries, we removed duplicate sequences (same day and location) from Nicaragua and Peru, yielding a final dataset of 436 genomes (including 66 genomes collected in Brazil between 1990 to 2013). Maximum likelihood phylogenies of the American DENV2 genomes (n = 670 and n = 436) were generated using PhyML(6) available through Seaview v.4.6.1,(7) using gamma-distributed among site rate heterogeneity and a general time reversible nucleotide substitution model. Root-to-tip divergence and temporal signal was evaluated using TempEst.(8) Georefenced and time-stamped phylogenies were constructed using a discrete phylogeographic approach as previously described.(9,10) In brief, countries were grouped into four geographic regions consisting of Brazil (n = 86), Central America and Mexico (n = 45), South America (n = 132) and Caribbean (n = 173). Inferred locations at each internal node and corresponding dated phylogenies trees were estimated using BEAST1.10.(9,10,11) MCMC convergence was inspected using Tracer.v1.7 and summary trees were generated using TreeAnnotator.(11)

TABLE III

GenBank accession numbers for sequences generated in this study

ID	GenBank Accession Number
4000	MN959474
4011	MN959475
4013	MN959483
4019	MN959476
4020	MN959482
4112	MN959478
4210	MN959477
4213	MN959480
4216	MN959485
4105	MN959479
4218	MN959481
126	MN959484
137	*
138	MN959469
140	MN959467
142	MN959468
143	MN959470
144	MN959472
145	MN959471
146	MN959473

*: parcial genome available on Github (https://github.com/CAD-DECentre/DENV2-PILOT).

REFERENCES

1. Quick J, Grubaugh N, Pullan ST, Claro IM, Smith AD, Gangavarapu K, et al. Multiplex PCR method for MinION and Illumina sequencing of Zika and other virus genomes directly from clinical samples. Nat Protoc. 2017; 12: 1261-76.
2. Clark K, Karsch-Mizrachi I, Lipman DJ, Ostell J, Sayers EW. GenBank. Nucleic Acids Res. 2016; 44: D67-72.
3. Katoh K, Asimenos G, Toh H. Multiple alignment of DNA sequences with MAFFT. Methods Mol Biol. 2009; 537: 39-64.
4. Larsson A. AliView: a fast and lightweight alignment viewer and editor for large datasets. Bioinformatics. 2014; 30: 3276-8.
5. Price MN, Dehal PS, Arkin AP. FastTree 2 - approximately maximum-likelihood trees for large alignments. PLoS One. 2010; 5: e9490.
6. Guindon S, Delsuc F, Dufayard JF, Gascuel O. Estimating maximum likelihood phylogenies with PhyML. Methods Mol Biol. 2009; 537: 113-37.
7. Gouy M, Guindon S, Gascuel O. SeaView version 4: a multiplatform graphical user interface for sequence alignment and phylogenetic tree building. Mol Biol Evol. 2010; 27: 221-4.
8. Rambaut A, Lam TT, Max Carvalho L, Pybus OG. Exploring the temporal structure of heterochronous sequences using TempEst (formerly Path-O-Gen). Virus Evol. 2016; 2: vew007.
9. Faria NR, Quick J, Claro IM, Thesé J, de Jesus JG, Giovanetti M, et al. Establishment and cryptic transmission of Zika virus in Brazil and the Americas. Nature. 2017; 546: 406-10.
10. Lemey P, Rambaut A, Drummond AJ, Suchard MA. Bayesian phylogeography finds its roots. PLoS Comput Biol. 2009; 5: e1000520.
11. Suchard MA, Lemey P, Baele G, Ayres DL, Drummond AJ, Rambaut A. Bayesian phylogenetic and phylodynamic data integration using BEAST 1.10. Virus Evol. 2018; 4: vey016.

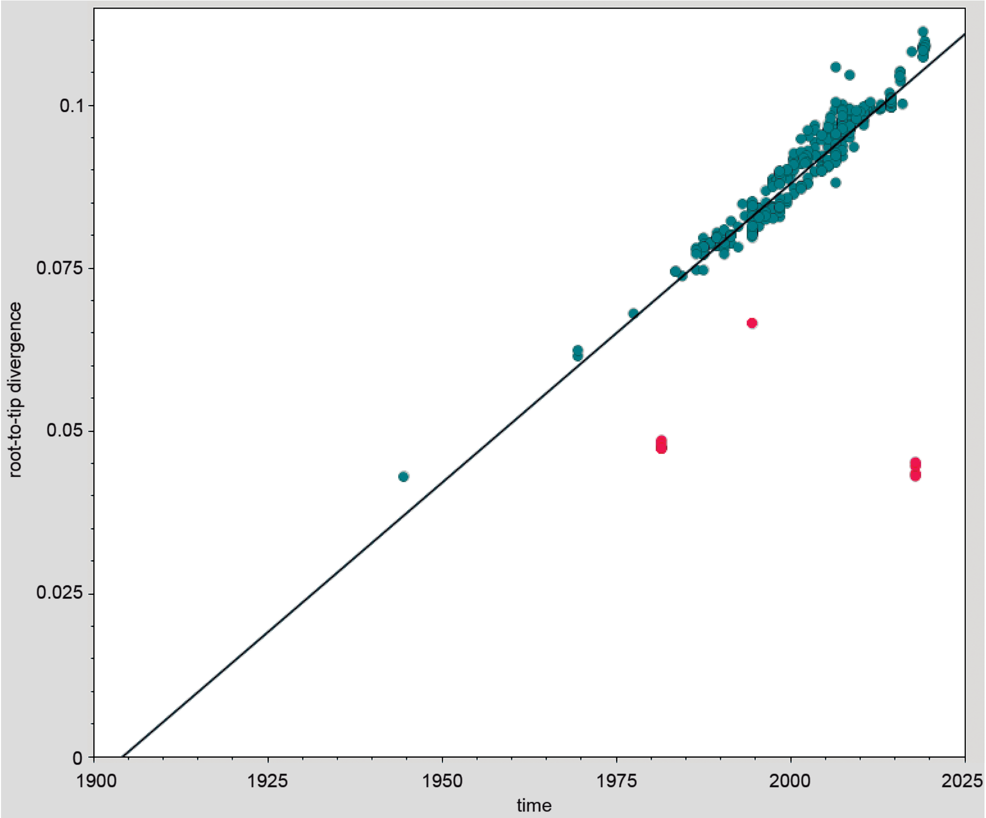

Fig. 1: root-to-tip regression of sequence sampling date against genetic divergence from the root of dengue virus (DENV) serotype 2 in the Americas.

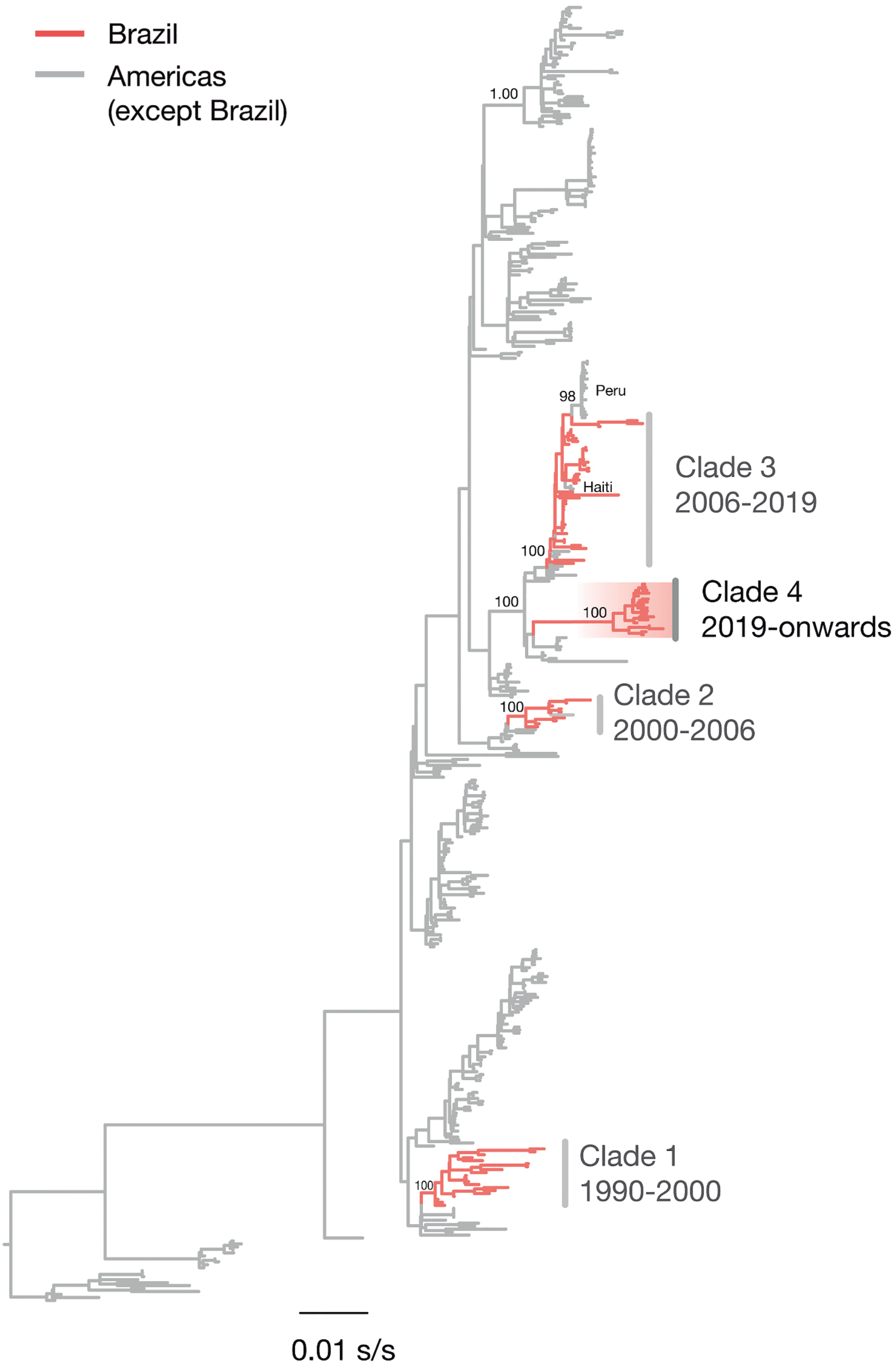

Fig. 2: maximum likelihood phylogeny of dengue virus (DENV) serotype 2 (n = 436) in the Americas. Tips (and nodes leading to) Brazilian strains are shown in red. New clade comprising isolates from 2019 collected in Araraquara and São José do Rio Preto (São Paulo State) are highlighted with a red gradient.
